# Five Weeks in a Trance

**Published:** 1881-03

**Authors:** 


### FIVE WEEKS IN A TRANCE.

Physicians in Newark, N. J., have been deeply interested lately in a curious case of hysteria in that city. For five weeks beginning in November, Miss Anna Ward, the sixteen-year-old step-daughter of Alexander Johnson, of the Mutual Benefit Life Insurance Company, lay in a trance. She lay quietly in her bed with her eyes sometimes open and sometimes shut, but recognizing no one, and never speaking. No sound escaped her, and it was evident she suffered no pain. There was a slight twitching of the eyelids, but little other movement. Physicians concluded that she was a victim of hysteria in an aggravated form, resulting from over study. The severest electric shocks caused not even the twitching of a muscle. After several days had passed Dr. O'Gorman, not knowing how long the trance would last, decided to administer liquid food artificially, as the patient could not swallow. About New Year's day she revived, and now she is able to ride out, and seems to be restored to health. While she was in the trance the physicians were satisfied that she was conscious, and proved it two or three times. Once Dr. Seguin said for a test : "She is a very pretty girl." Immediately she blushed. She says she was conscious, but had only one thought, and that a horrible one. She feared constantly that the physicians would pronounce her dead, and she would be buried alive. She had no physical pain, but this dread was agonizing. In vain did she try to speak. She could not even move her lips.

---

THERE is a right and a wrong way of rubbing a man's mind as well as a cat's back.—*Lavater.*

---

IT is difficult to realize the enormous power of the bright speck, Jupiter, shining so quietly in the sky. A writer has shown that the power which the sun has to put forth to hold Jupiter in his orbit is equal to the combined strength of 170,000,000 bars of solid steel, each one a mile in diameter. Jupiter's pull upon the earth, according to the same authority, is equal to the strength of 23,000,000,000 bars of steel, each of them one foot in diameter. So, if the mere power of gravity were all that were required to make Jupiter's approach dangerous to the earth, evidently he is not lacking in the power. But no one need fear that the sisterhood of worlds which acknowledge the dominion of the sun will prove equally destructive.

